# Dentistry and Medicine

**Published:** 1887-02

**Authors:** Herbert M. King


### DENTISTRY AND MEDICINE.

BY HERBERT M. KING, M. D.

Dr. Norman Kingsley's letter in the *Medical Record* for November 20th, and later, his discourse before the First District Dental Society, at a special meeting of that body, express a sentiment which will inevitably meet with much opposition among dental practitioners. The object of this movement, of which Dr. Kingsley seems to be the leader in the First District Society, is the establishment of an independent profession in dental surgery, throwing off all allegiance to medicine and creating a relationship between the two as distant as that between law and theology. Among the more forcible arguments brought forward in favor of this movement, is the citation of the old fact that the medical profession at one time did refuse to acknowledge the dental specialty. The force of this argument, however, lies in the sensitive consciousness of certain representatives of a specialty, the youth of which forbids, for the present at least, a fair estimate of its standing. It must not be forgotten that at the period of this occurrence there was a powerful opposition to the branching out of all exclusive specialties from a general practice.

To-day the condition of things is different. Eminent authors and members of the profession at large who have given the question a thought, almost universally acknowledge dental surgery as a specialty of that branch of science which treats of disease, and the methods employed for its relief. And if to-day dentists would warrant the action by their support, there is scarcely a medical institution in the country which would not place in its faculty a chair of Oral and Dental Surgery (since Dr. Kingsley insists upon distinguishing between the two), and provide conveniences for the practical teaching of the same.

Dr. Kingsley's arguments favoring the evolution of Dentistry into an independent profession might as appropriately be applied to ophthalmology, orthopœdia, or almost any recognized specialty, but particularly orthopœdic surgery, for, says Dr. Kingsley: "The predominating feature and characteristic of dentistry, that which removes it further than all else combined from medicine, is the mechanical nature of its methods." It seems to me that the methods of treatment adopted in orthopœdia partake of mechanics and mechanical principles quite as much as do they in dental surgery. Yet who would question the fact that orthopœdic surgery is a specialty of Medicine, and surely, least of all, its own practitioners?

It is highly probable that other specialties have more to do with the etiology of the several morbid conditions which they treat, than does the dental specialty with its peculiar affections. This fact, however, arises from one of two sources: either the knowledge of causes of morbid conditions in the teeth cannot aid in the treatment of the same, or the education of the dentist is not inclusive enough to enable him to appreciate the connection between cause and effect, and if this latter be the fact, a more general knowledge of possible constitutional conditions is at least very desirable for the thorough understanding and practical usage of this relationship in cases under the direction of the dental specialist. And yet, Dr. Kingsley would have us believe that a general medical education is a superfluous and unnecessary accomplishment in the competent and efficient dental specialist. Indeed, he says that Medicine has little or nothing in common with Dentistry. The fallacy of this statement is too apparent to call for a labored argument, and it needs but a momentary consideration to convince the thoughtful of the unquestionable fact that Medicine is the foundation out of which the dental specialty properly springs—not as "a ballast for the higher flight of the medical kite," but as an honorable and needful branch, the thorough understanding and successful practice of which necessarily monopolizes the attention of the specialist and alone bars him from the pursuit of a more general practice.

The student, realizing this fact and intending to follow the life of the specialist, turns his principal attention and study to the immediate fields of his practical requirements, concentrating his energies, as it were, rather than scattering them. Together with the general student he studies the preliminaries in anatomy, physiology, chemistry and histology. So far the education of the two is alike, but the special student directs his subsequent efforts to the study of that portion of practice having mostly to do with his chosen specialty; that portion of materia medica and therapeutics and that of surgery best adapted to the fulfillments of his future requirements, and in the end his education, even though attained in a dental college, is really medical, his course being derived from and remaining a part of medical science, and only differing from that science in that it elaborates a part rather than attempts a whole.

In full contemplation of these facts it is impossible to regard dental surgery in the light of an unique profession, since it has, in common with general medicine, such a vast deal absolutely essential to its existence. Its literature is not independent, since it is but a digested and enlarged study of that portion of medical literature having most practical interest for the dentist. Dentistry cannot be divorced from Medicine and regarded as an independent profession, since it has wholly to do with morbid conditions in the living body and the methods employed for the relief thereof. In brief, dental surgery is but the perfected specialty rather than the entirety in itself.

Follow the history of a disease which from its peculiar nature comes to the dental specialist for treatment. Do we not find presenting, as in disease of any other member, an etiology, a pathology, a train of symptoms, a diagnosis, a prognosis, and the treatment indicated? And are not the pathological changes attendant upon a carious tooth or divitalized pulp intimately associated in character with pathological changes elsewhere, and best comprehended by the mind that has not wholly disregarded general pathology?

The practiced hand, with but a little mechanical ingenuity behind it, may be able to prepare and fill a dental cavity as successfully as would the graduated specialist, but I am confident that no thoughtful and educated person would wish to place even the most trivial affections of his mouth in the charge of the man lacking the ability to appreciate the whole history and probable future of the case, and whose only virtue is his mechanical skill.

If Dentistry is to be pursued as a trade, by all means separate it from Medicine, and waste no time in studying that science. But if it is to be regarded in a higher light and practiced accordingly, it cannot properly be considered as other than a specialty of that grand division of science, the object of which is the cure of disease and the preservation of health.

